# Long noncoding RNA LINC00284 facilitates cell proliferation in papillary thyroid cancer via impairing miR-3127-5p targeted E2F7 suppression

**DOI:** 10.1038/s41420-021-00551-8

**Published:** 2021-06-26

**Authors:** Bin Zhou, Yugang Ge, Qing Shao, Liyi Yang, Xin Chen, Guoqin Jiang

**Affiliations:** 1 Department of General Surgery, The Second Affiliated Hospital of Soochow University, Suzhou, 215000 Jiangsu Province China; 2 Department of Thyroid and Breast Surgery, The Affiliated Jiangyin Hospital of Southeast University Medical College, Wuxi, 214000 Jiangsu Province China

**Keywords:** Long non-coding RNAs, Head and neck cancer

## Abstract

Accumulating evidence has suggested that long noncoding RNAs (lncRNAs) exert crucial modulation roles in the biological behaviors of multiple malignancies. Nonetheless, the specific function of lncRNA LINC00284 in papillary thyroid cancer (PTC) remains not fully understood. The objective of this research was to explore the influence of LINC00284 in PTC and elucidate its potential mechanism. The Cancer Genome Atlas (TCGA), gene expression omnibus (GEO) datasets were used to analyze LINC00284 expression differences in thyroid cancer and normal samples, followed by the verification of qRT-PCR in our own PTC and adjacent non-tumor tissues. The impacts of LINC00284 on PTC cell growth were detected in vitro via CCK-8, colony formation, EdU assays, and in vivo via a xenograft tumor model. Bioinformatics analyses and biological experiments were conducted to illuminate the molecular mechanism. We found that LINC00284 expression was remarkably increased in PTC tissues and its overexpression was closely correlated with larger tumor size. In addition, silencing LINC00284 could effectively attenuate PTC cell proliferation, induce apoptosis and G1 arrest in vitro, as well as suppress tumorigenesis in mouse xenografts. Mechanistic investigations showed that LINC00284 acted as a competing endogenous RNA (ceRNA) for miR-3127-5p, thus resulting in the disinhibition of its endogenous target E2F7. In short, our findings indicated that LINC00284–miR-3127-5p–E2F7 axis exerted oncogenic properties in PTC and may offer a new promising target for the diagnosis and therapy of PTC.

## Introduction

Thyroid cancer (TC) represents the most frequent endocrine malignancy stemming from thyroid follicular cells or parafollicular cells and the fifth most prevalent cancer for women, whose incidence ranks the ninth in global cancer, with about 57,000 new cases annually worldwide [1]. Papillary thyroid cancer (PTC) is the main histological type among all thyroid tumors, usually characterized by indolent biological behaviors, accounting for more than 80% of the total [2]. Through thyroidectomy, radioiodine, or TSH suppression therapy [3], most PTC patients can be cured and possess a favorable 5-year survival rate of more than 90% [4]. However, there was a subset of patients suffering from lymph node or distant organ metastasis, leading to poor prognosis [5]. So, there is a necessary to clarify the molecular mechanism underlying PTC for exploring new effective therapeutic strategies so as to meet clinical needs.

Recent whole-genome and transcriptome researches have revealed that only ~2% human genome can encode proteins, while >75% of them are transcribed into noncoding RNAs, including the vast majority of long noncoding RNAs (lncRNA) [6, 7]. LncRNAs are defined as a category of transcripts comprising over 200 nucleotides once considered as transcriptional noise [8]. Accumulating evidence has expounded that lncRNAs exert crucial roles in modulating gene expression in various aspects, such as competitive endogenous RNAs (ceRNAs, that is miRNA sponges) [9], epigenetic programming [10], mRNA alternative splicing [11], protein activities [12], and alteration of protein localization [13]. A large portion of lncRNAs are dysregulated or abnormally expressed to contribute various human diseases, containing malignancies [14, 15]. LINC00284, as a member of lncRNAs, locating in 13q14.11, was regulated by ALDH1A3 to involve in differentiation and catabolic processes in triple-negative breast cancer [16]. In ovarian cancer, Ruan et al. identified that silencing LINC00284 could suppress the transcriptional expression of MEST via NF-ΚB1, in turn, attenuated angiogenesis [17]. Besides, hepatocellular carcinoma patients with high expression of LINC00284 possessed poor recurrence-free survival [18]. However, the biological function of LINC00284 in PTC remains unclear.

In this current study, datasets from the Cancer Genome Atlas (TCGA) and gene expression omnibus (GEO) databases were firstly applied to evaluate LINC00284 expression in TC samples and normal samples. After that, collecting our own hospital’s PTC samples and cells, we further investigate its clinical significance and specific biological function, as well as revealing the potential ability to act as miRNA sponges through a series of bioinformatics analyses and experimental verification.

## Results

### LINC00284 is highly expressed in PTC

**Fig. 1:**
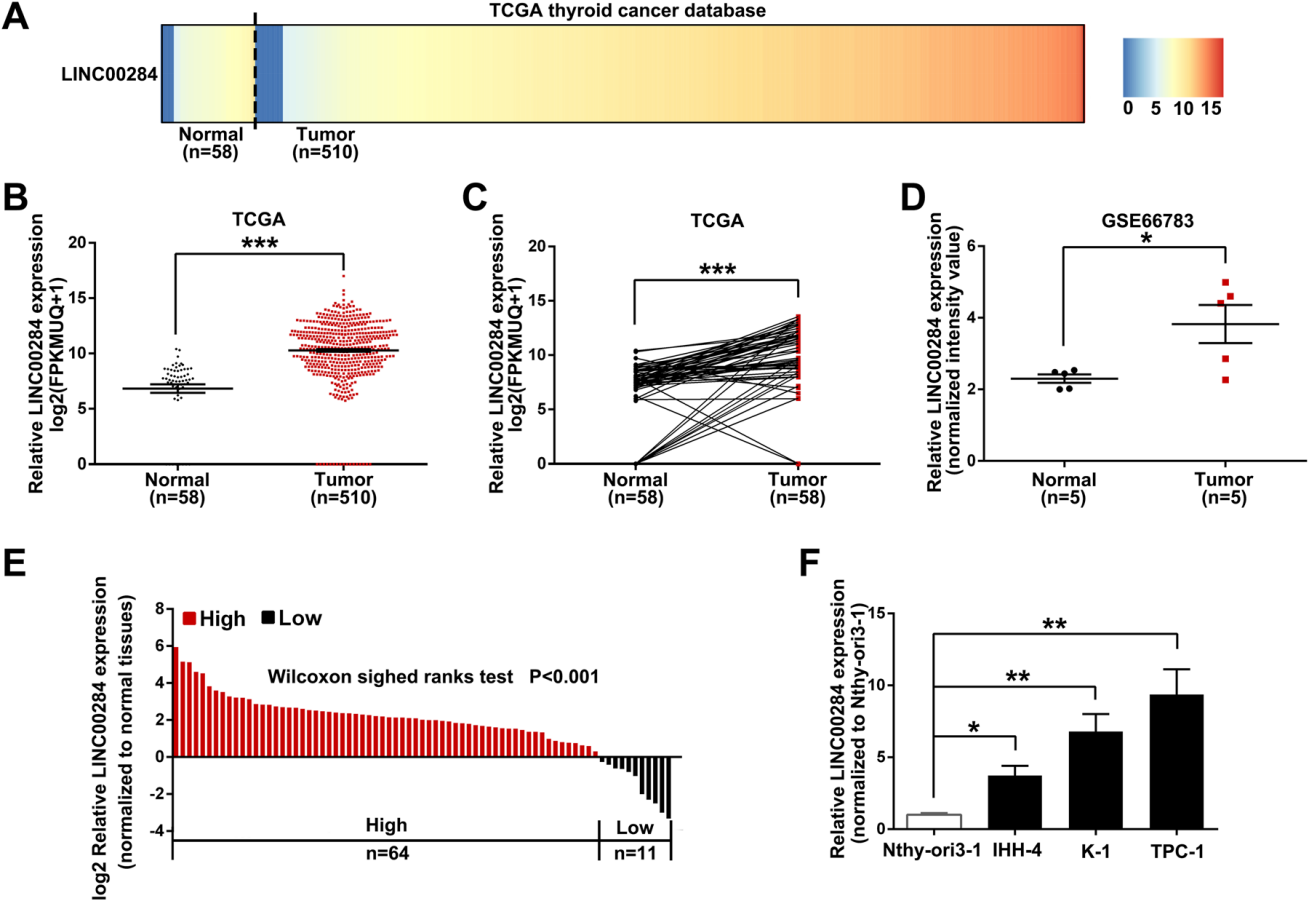
LINC00284 is highly expressed in papillary thyroid cancer (PTC). **A** Heat map analysis of LINC00284 expression in TC tissues and normal tissues from TCGA. **B** *T*-test was conducted to assess the differential expression of LINC00284 in TC samples and normal samples using TCGA data. **C** Relative LINC00284 expression in paired TC samples and normal samples in TCGA. **D** The data of GEO was extracted to evaluate LINC00284 expression in paired TC specimens and normal specimens. **E** qRT-PCR analyses of LINC00284 expression in PTC tissues and adjacent noncancerous tissues from our own hospital. **F** qRT-PCR was conducted to analyze LINC00284 expression in the normal thyroid follicular epithelial cell (Nthy-ori3-1) and PTC cells. Values indicated mean ± standard errors. *P < 0.05, **P < 0.01, and ***P < 0.001.

To figure out whether LINC00284 was abnormally expressed in TC, we first extracted the RNA sequencing data of 510 TC tissues and 58 normal tissues from TCGA to take heat map analysis, and observed that LINC00284 expression was increased in a large proportion of cancer patients (Fig. 1A). The results of the further *t*-test indicated that LINC00284 expression levels were remarkably upregulated both in unpaired and paired cancer samples (Fig. 1B and C). We also found the same trend via analyzing the other published profiling data (GSE66783) of GEO (Fig. 1D). Then we confirmed the RNA-seq results by examining the expression of LINC00284 in 75 paired PTC specimens and adjacent normal specimens, and discovered that PTC tissues displayed higher LINC00284 expression (Fig. 1E). Meanwhile, the expression levels of LINC00284 were higher in PTC cells (IHH-4, K-1, and TPC-1) than that in normal thyroid follicular epithelial cells (Nthy-ori3-1) (Fig. 1F).

Subsequently, to explore the relationship between LINC00284 expression and the clinical features of PTC patients, we classified the patients as high or low expression group based on the median value of LINC00284 expression. As shown in Table 1, higher LINC00284 expression in PTC tissues was markedly related to larger tumor size (*P* = 0.014), but there was no obvious association between LINC00284 expression and gender, age, extrathyroidal extension, lymph node metastasis, multifocality, and TNM stage.

**Table 1:** Correlation between LINC00284 expression and clinicopathological features in papillary thyroid cancer.

Characteristics	Number	LINC00284 expression		*P*-value
		High	Low	
Gender				
Male	31	14	17	0.423
Female	44	24	20	
Age (years)				
<45	44	21	23	0.544
≥45	31	17	14	
Extrathyroidal extension				
Yes	14	9	5	0.258
No	61	29	32	
Tumor size (cm)				
≤2	53	22	31	**0.014**
>2	22	16	6	
Lymph node metastasis				
Yes	39	23	16	0.134
No	36	15	21	
Multifocality				
Yes	15	10	5	0.166
No	60	28	32	
TNM stage				
I + II	57	27	30	0.309
III + IV	18	11	7	

Bold value indicates statistically significant difference.

### LINC00284 promotes PTC cell proliferation

**Fig. 2:**
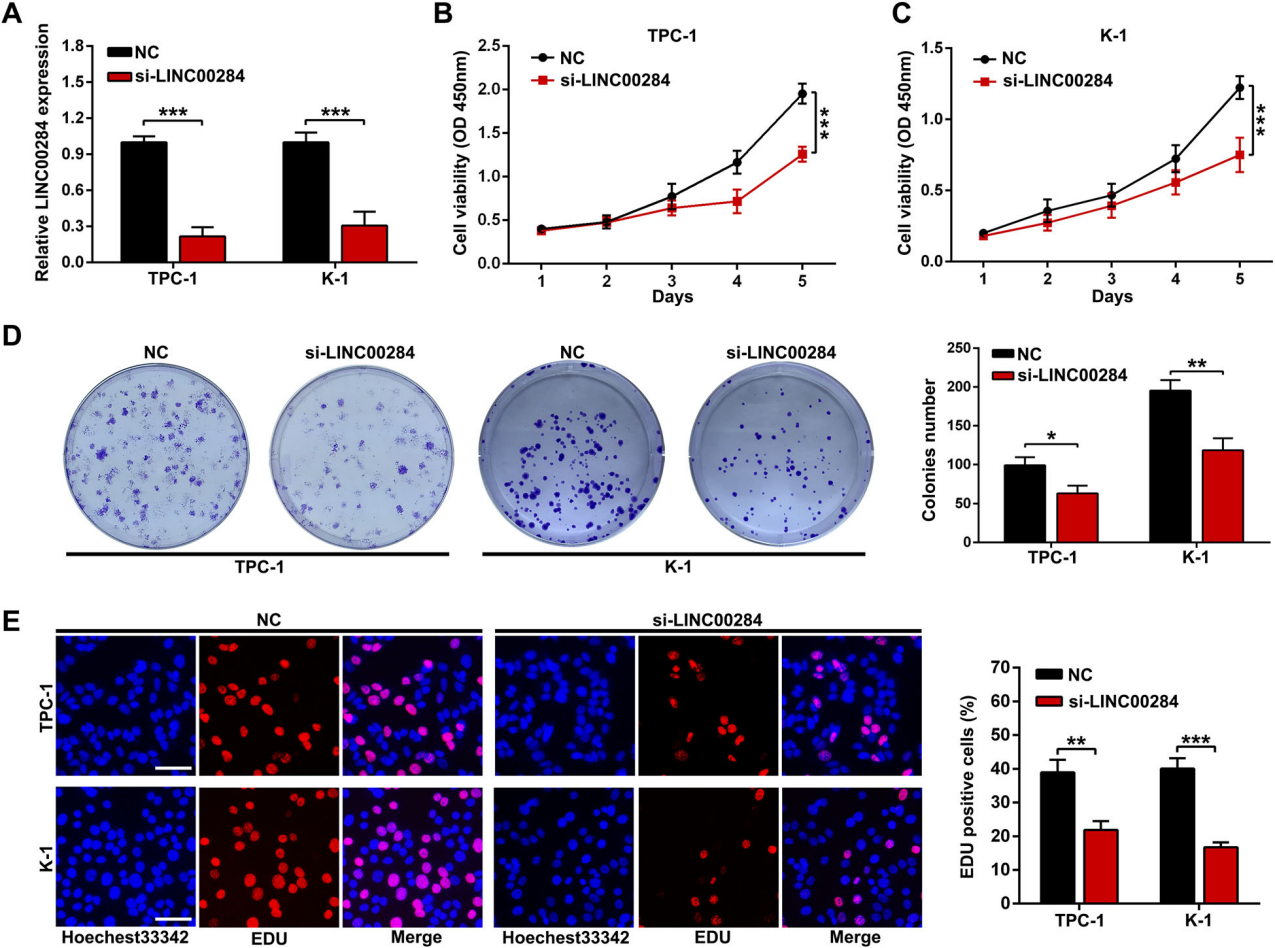
Impacts of LINC00284 on PTC cell proliferation in vitro. **A** LINC00284 expression in TPC-1 and K-1 treated by si-LINC00284 or si-NC was detected by qRT-PCR. **B** CCK8 experiments were adopted to assess the viability of si-LINC00284 treated PTC cells. **C** Colony formation assays were used to examine the growth of PTC cells after transfection. **D** The proliferation ability of PTC cells transfected with si-LINC00284 was analyzed by EdU staining assays (scale bar: 100 μm). Values indicated mean ± standard deviation. *P < 0.05, **P < 0.01, and ***P < 0.001.

The TPC-1 and K-1 cells, presenting relatively higher expression of LINC00284, were selected to knockdown LINC00284 with siRNA. After 48 h, the results of qRT-PCR showed the satisfactory transfection efficiency that LINC00284 expression was obviously downregulated in the si-LINC00284 group than that in NC group for TPC-1 or K-1 cells (Fig. 2A). Considering the association between LINC00284 expression and tumor size, we investigated the influences of LINC00284 on PTC cell proliferation. The cell growth curves from CCK-8 assays revealed that silencing LINC00284 could cause a decrease in the proliferation of TPC-1 and K-1 cells compared with the NC group (Fig. 2B and C). The colony-formation assay also identified that downregulating LINC00284 suppressed the proliferation of these cells (Fig. 2D). Similarly, we found that knockdown of LINC00284 had a remarkable inhibition on PTC cell growth through the EdU assay (Fig. 2E).

### Silencing LINC00284 induces apoptosis, G1 arrest of PTC cells, and attenuates tumorigenesis in vivo

**Fig. 3:**
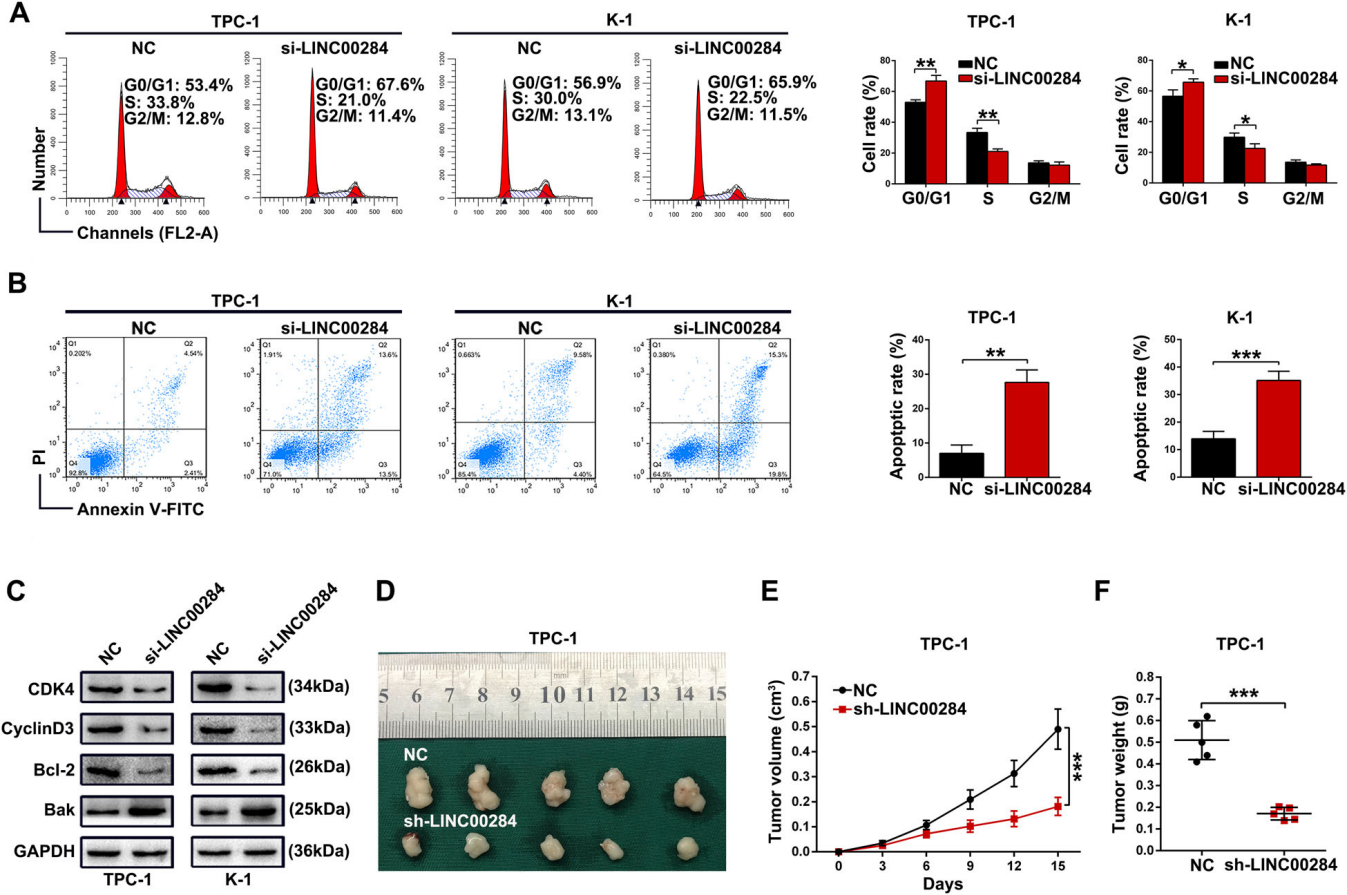
Effects of LINC00284 silencing on PTC cells cycle and apoptosis and PTC tumorigenesis in vivo. **A** Flow cytometric analysis of TPC-1 and K-1 cells transfected with siRNAs or control about cell cycle arrest. **B** The apoptotic rates of transfected cells were examined by flow cytometry. **C** The expression levels of cell cycle-related and apoptosis-related proteins after silencing LINC00284. **D** The stable LINC00284 silencing TPC-1 cells were utilized for in vivo experiments. Tumor sizes of the harvested xenografts from shRNAs and control groups were shown. **E** After injection, tumor volumes were calculated every three days and growth curves were drawn. **F** The weight of xenografts was measured. Values indicated mean ± standard deviation. *P < 0.05, **P < 0.01, and ***P < 0.001.

As we all known, cell cycle and apoptosis were two common elements influencing tumor cell growth ability, thus we performed the flow-cytometric assay to assess the impacts of LINC00284 on these two properties. The results suggested that inhibiting LINC00284 in TPC-1 and K-1 cells brought about increased cell proportion in G0/G1 phase while reduced cells in the S phase (Fig. 3A). In comparison with the NC group, the apoptosis of the si-LINC00284 group was found to be significantly accelerated (Fig. 3B). In addition, in line with the cell cycle progression data, cells transfected by si-LINC00284 expressed obviously lower levels of G1–S-phase checkpoint proteins, including CDK4 and Cyclin D3. After silencing LINC00284, the expression level of pro-apoptosis protein like Bak was significantly upregulated while anti-apoptosis proteins like Bcl-2 were strongly decreased (Fig. 3C). To further illustrate whether LINC00284 affected tumor growth in vivo, we inoculated subcutaneously TPC-1 cells treated by sh-LINC00284 or control vector into female nude mice. It’s clear that tumor volume and weight decreased after inhibiting LINC00284 expression in TPC-1 cells (Fig. 3D–F). Immunohistochemical analysis revealed sh-LINC00284 group displayed reduced expression of Ki-67 and upregulation of Cleaved Caspase-3 expression compared with the negative control (Supplementary Fig. 1).

### LINC00284 serves as a ceRNA sponging miR-3127-5p in PTC

**Fig. 4:**
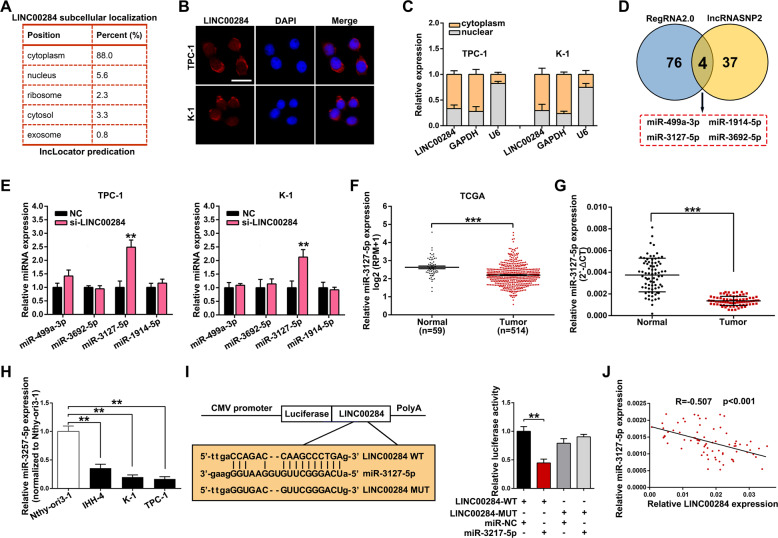
LINC00284 serves as a ceRNA sponging miR-3127-5p. **A** The online tool lncLocator was utilized to forecast the subcellular localization of LINC00284. **B** RNA FISH assay was performed to analyze the location of LINC00284 in the nuclear (blue) and cytoplasm (red) fractions in TPC-1 and K-1 cells (scale bar: 50 μm). **C** The subcellular fractionation assay verified the location of LINC00284 in TPC-1 and K-1 cells. **D** Bioinformatics analyses screened the miRNAs binding with LINC00284 by RegRNA 2.0 and lncRNASNP2. **E** The expression levels of four speculated miRNAs via qRT-PCR after inhibiting LINC00284. **F** Relative expression of miR-3127-5p in TC samples in comparison to normal samples was assessed by applying TCGA dataset. **G**, **H** MiR-3127-5p expression was detected by qRT-PCR in PTC tissues and cells. **I** Dual-luciferase reporter assay for verification of the binding between LINC00284 and miR-3127-5p. **J** Correlational analyses of the expression of LINC00284 and miR-3127-5p in 75 PTC samples. Values indicated mean ± standard deviation. **P < 0.01 and ***P < 0.001.

Recently, increasing evidence has illuminated that plenty of lncRNAs could act as ceRNAs to sponge miRNAs, modulating the expression of target genes at the post-transcriptional level. Firstly, using the online tool “lncLocator”, we predicted that most LINC00284 existed in the cytoplasm of cells (Fig. 4A). Then the FISH and subcellular fractionation assay were conducted and confirmed the above outcome that most of LINC00284 located in the cytoplasm of PTC cells, suggesting that it might exert its functions as ceRNA (Fig. 4B and C). The programmes, such as lncRNASNP2 and RegRNA 2.0, were carried out for predicting potential miRNAs binding to LINC00284, in result, four miRNAs were selected (miR-499a-3p, miR-1914-5p, miR-3127-5p, and miR-3692-5p) (Fig. 4D). We then investigated the impacts of LINC00284 knockdown on the expression of these four miRNAs via qRT-PCR assay and observed that miR-3127-5p expression level was dramatically upregulated after inhibiting LINC00284 in TPC-1 or K-1 cells. And given that miR-3127-5p has been demonstrated to serve as a tumor suppressor in various carcinomas, thus we finally chose it for further investigation. Likewise, we used the TCGA database to evaluate miR-3127-5p expression and found that TC tissues possessed lower expression of miR-3127-5p when compared with normal tissues (Fig. 4F), which was also subsequently verified in our own collected PTC specimens and adjacent non-cancer specimens (Fig. 4G). Furthermore, the expression of miR-3127-5p in PTC cells was lower than that in normal thyroid follicular epithelial cells (Fig. 4H). Figure 4I exhibited the putative binding sequence between LINC00284 and miR-3127-5p from lncRNASNP2. After that, the luciferase reporters including the mutated LINC00284 sequence which changed the binding sites of miR-3127-5p were designed, and transduced into HEK-293T cells together with miR-3127-5p mimics or control. As anticipated, we found that the mutation of LINC00284 abolished the inhibition of luciferase activity mediated by miR-3127-5p. Besides, there was a remarkable inverse association (*R* = −0.507, *P* < 0.001) between LINC00284 and miR-3127-5p levels when making qRT-PCR analyses of 75 PTC tissues (Fig. 4J).

### Knockdown of LINC00284 inhibits tumor proliferation via miR-3127-5p

**Fig. 5:**
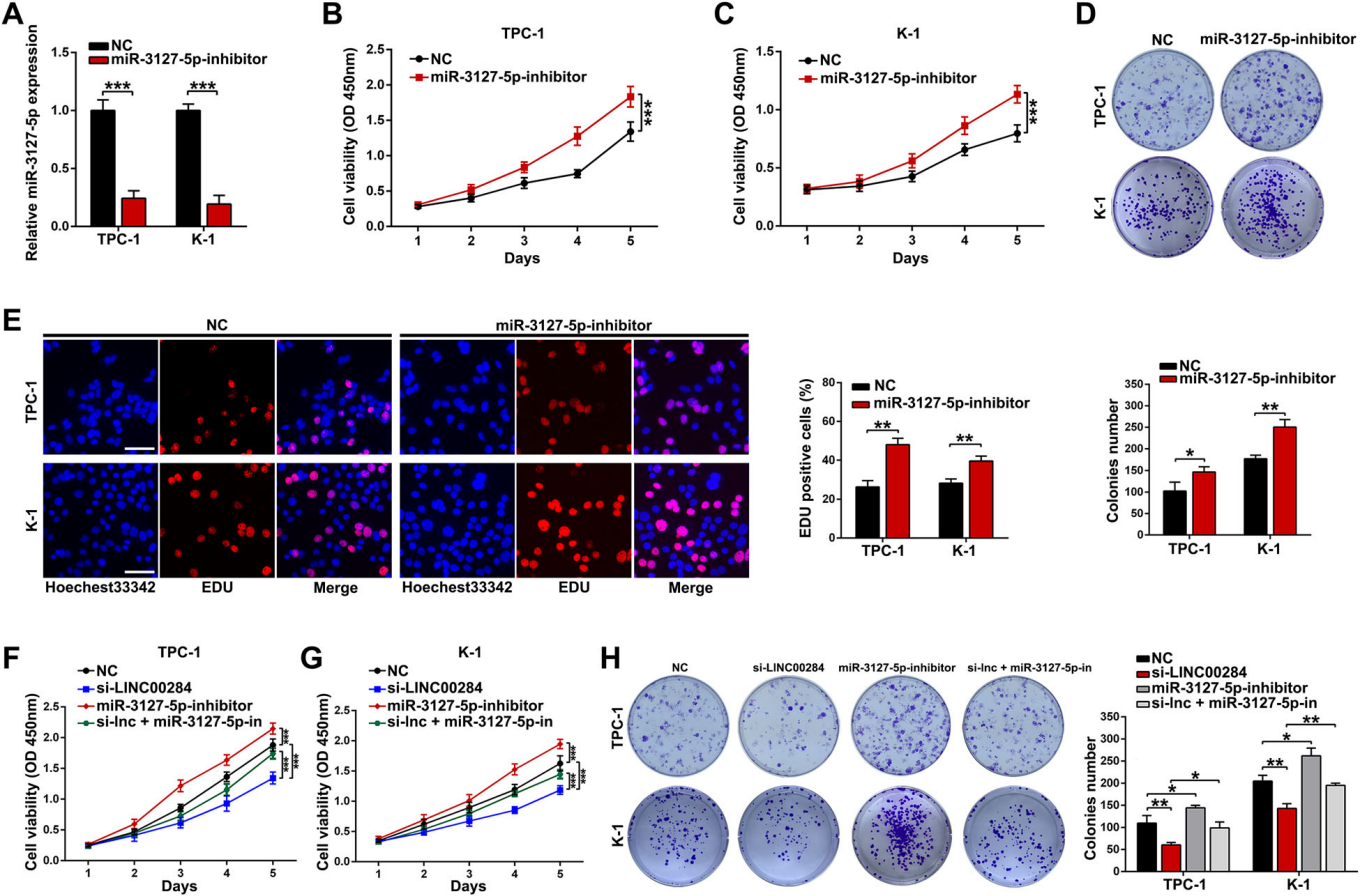
Knockdown of LINC00284 inhibits tumor proliferation via suppressing miR-3127-5p. **A** After being treated with miR-3127-5p inhibitor or control miRNA, the transfection efficiency was detected with qRT-PCR in TPC-1 or K-1 cells. B-E, CCK8, colony formation, and EdU experiments were adopted to measure the proliferation viability of TPC-1 and K-1 transfected miR-3127-5p inhibitor (scale bar: 100 μm for EdU assay). **F**–**G** The growth ability of TPC-1 and K-1 cells after cotransfection with si-LINC00284, miR-199a-5p inhibitor, or control miRNA were determined by CCK8 and colony formation assays. Values indicated mean ± standard deviation. *P < 0.05, **P < 0.01, and ***P < 0.001.

Although it has been proven that miR-3127-5p inhibited tumor cell development and progression in various malignancies, its role in PTC remained unknown. So we transfected miR-3127-5p inhibitor into TPC-1 and K-1 cells to identify whether it plays a tumor-suppressive role in PTC, and Fig. 5A showed the effective silencing efficiency. Then we conducted the CCK-8, colony formation, and EdU assays, observing the significant enhancement of cell growth and colony formation ability (Fig. 5B–E). Meanwhile, to reveal whether the influences of LINC00284 on PTC cells were mediated by miR-3127-5p, si-LINC00284, and miR-3127-5p inhibitor were cotransfected into TPC-1 and K-1 cells. As a result, we found that the attenuation of cell proliferation arisen from LINC00284 knockdown could be partially rescued by miR-3127-5p inhibitor via CCK-8 and colony formation assays (Fig. 5F–H).

### E2F7 is the target of miR-3127-5p and indirectly modulated by LINC00284

**Fig. 6:**
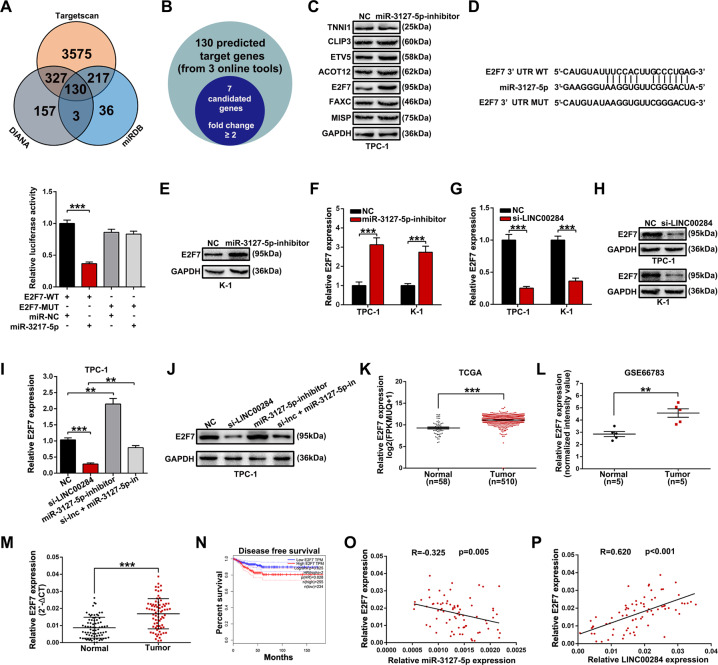
E2F7 is a target of miR-3127-5p and is repressed by downregulating LINC00284. **A** Three bioinformatics tools (Targetscan, DIANA, and miRBD) were used to predict target genes binding with miR-3127-5p. **B** Seven mRNAs from the total predicted genes were selected according to the following criterion (its expression level in TC tissues was higher than that in normal tissues, fold change ≥ 2) by analyzing TCGA data. **C** Western blot assays were performed to determine the protein levels of seven candidate target mRNAs after TPC-1 cells being treated by miR-3127-5p inhibitor. **D** Dual-luciferase reporter assay was performed to confirm the binding between miR-3127-5p and E2F7. **E** The protein level of E2F7 in K-1 cells following miR-3127-5p inhibitor. **F** E2F7 mRNA levels were analyzed by qRT-PCR in TPC-1 and K-1 cells treated by miR-3127-5p inhibitor. **G**, **H** E2F7 mRNA and protein levels in TPC-1 and K-1 cells treated by si-LINC00284. **I**, **J** Evaluation of E2F7 mRNA and protein levels through rescue assays that cotransfected with si-LINC00284 and miR-3127-5p inhibitor in TPC-1 cells. **K**, **L** Relative expression of E2F7 in TC samples and normal samples using TCGA and GEO data. **M** qRT-PCR analysis of E2F7 expression from PTC tissues and adjacent noncancerous tissues of our own hospital. **N** The online GEPIA tool was applied to draw the disease-free survival curve. **O**, **P** Correlation analyses of the expression of E2F7 and miR-3127-5p, the expression of E2F7 and LINC00284 in 75 PTC tissues. Values indicated mean ± standard deviation. **P < 0.01 and ***P < 0.001.

The online bioinformatics tools such as Targetscan, DIANA, and miRDB were utilized to search the downstream targets of miR-3127-5p, and the final intersection of these three datasets was 130 genes (Fig. 6A). Combining the TCGA database, we sorted out seven mRNAs with markedly higher expression in TC specimens than in normal specimens as candidate target mRNAs (fold change ≥ 2, *P* ≤ 0.05) (Fig. 6B). Next, we transfected miR-3127-5p inhibitor into TPC-1 cells and applied western blot experiments to detect the protein levels of the seven predicted target mRNAs. The results indicated that E2F7 protein level was upregulated and the change was most obvious, suggesting it is likely to be the target mRNA of miR-3127-5p (Fig. 6C). Similarly, we then conducted dual-luciferase reporter assay mentioned above and found that the mutation of E2F7 abolished the inhibition of luciferase activity mediated by miR-3127-5p (Fig. 6D). Combined with the previous Fig. 6C, further qRT-PCR and western blot analyses showed that miR-3127-5p inhibition could lead to a dramatical increase of E2F7 expression both at protein and mRNA level for TPC-1 and K-1 cells (Fig. 6E and F).

In order to validate the ceRNA network between LINC00284, miR-3127-5p, and E2F7, we silenced LINC00284 expression in TPC-1 or K-1 cells and discovered both mRNA and protein expression levels of E2F7 were markedly reduced (Fig. 6G and H). The above outcomes reminded us of LINC00284 modulating E2F7 expression through competitively binding to miR-3127-5p. Furthermore, miR-3127-5p inhibition could effectively reverse the decrease of E2F7 mRNA or protein expression driven by si-LINC00284 in TPC-1 cells (Fig. 6I and J). The TCGA and GSE66783 datasets displayed TC tissues possessed significantly higher expression of E2F7 than normal tissues (Fig. 6K and L), in keeping with the detected values in PTC samples and adjacent normal samples from our own hospital (Fig. 6M). The GEPIA bioinformatics tool predicted that TC patients with E2F7 high expression had shorter disease-free survival (Fig. 6N). Finally, a negative relation between miR-3127-5p and E2F7 mRNA expression was uncovered in 75 PTC samples (*R* = −0.325, *P* = 0.005), while a positive association between LINC00284 and E2F7 mRNA expression was found (*R* = −0.620, *P* < 0.001) (Fig. 6O and P).

### E2F7 promotes PTC cell growth

**Fig. 7:**
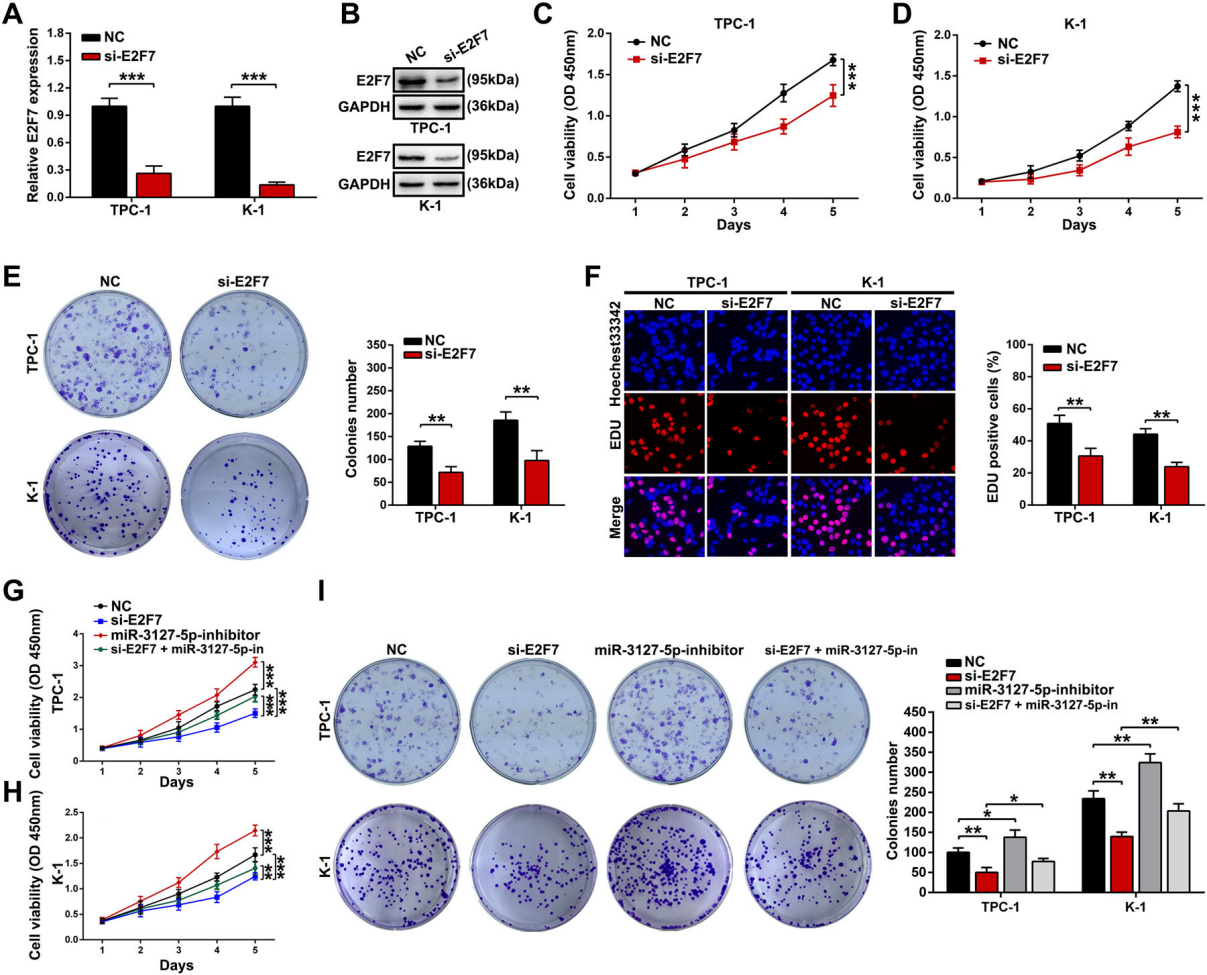
Influences of E2F7 on PTC cells growth in vitro. **A**, **B** E2F7 mRNA and protein levels were detected by qRT-PCR and western blot assays in E2F7 silencing TPC-1 and K-1 cells. **C** CCK8, colony formation, and EdU experiments were carried out to assess the proliferation viability of TPC-1 and K-1 cells transfected si-E2F7 or control siRNA (scale bar: 100 μm for EdU assay). **D** After cotransfection with si-E2F7, miR-3127-5p inhibitor, CCK8, and colony formation assays were used to draw and examine the growth curves and colony formation ability of TPC-1 and K-1 cells. Values indicated mean ± standard deviation. *P < 0.05, **P < 0.01, and ***P < 0.001.

Guo et al. reported that miR-30a targeted E2F7, inhibiting PTC cell progression. However, their study only proved that E2F7 was the target gene of miR-30a, which didn’t preliminarily explore its biological functions in PTC cells. So, we transfected E2F7 siRNA into TPC-1 or K-1 cells to knockdown its expression and verified it using qRT-PCR and western blot assays (Fig. 7A and B). We carried out the CCK-8, colony formation, and EdU incorporation experiments and found that downregulating E2F7 dramatically suppresses PTC cell proliferation (Fig. 7C–F). Moreover, the CCK-8 and colony formation assays revealed that inhibition of PTC cell growth induced by the silence of E2F7 was partially reversed by cotransfection with a miR-3127-5p inhibitor (Fig. 7G–I).

## Discussion

Mounting studies suggested that numerous lncRNAs were the key modulators of various tumor biologic processes, including proliferation, metastasis, glycolysis, angiogenesis, stem cell activity, and so on [19–21]. Researchers have gathered increasing attention for years to explore the pathogenesis underlying lncRNAs mediated cancer progression. Several lncRNAs have been verified to participate in PTC occurrence and development. For instance, Wu et al. determined that lncRNA SNHG15 functioned as a sponge of miR-200a-3p to modulate the YAP1-Hippo signaling pathway, influencing PTC cell growth and migration [22]. The G allele of rs619586 could remarkably reduce MALAT1 expression to promote PTC apoptosis, which was recognized a protective factor of PTC susceptibility [23]. Other reports also illuminated high expression of ABHD11-AS1, FOXD2-AS1, and CCAL portended poor clinical outcome of PTC sufferers [24–26]. Till now, the roles of LINC00284 in breast cancer [16], ovarian cancer [17], gastric cancer [27], and hepatocellular carcinoma [18] have been preliminarily investigated, while its biological effects in PTC weren’t illustrated.

**Fig. 8:**
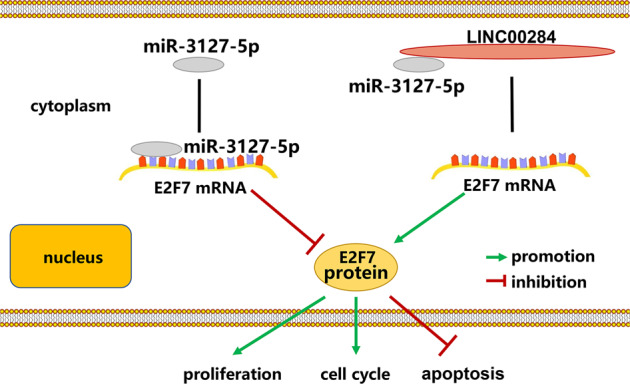
Mechanism model of lncRNA LINC00284 involved in PTC. LINC00284 can competitively bind to miR-3127-5p, liberating E2F7 mRNA transcripts and upregulating E2F7 expression, thus facilitates PTC cell proliferation, cell cycle progression, and inhibiting apoptosis.

In this present study, our prediction from TCGA and GEO databases, as well as qRT–qPCR analyses from our own PTC samples and cells, indicated markedly high LINC00284 expression in PTC tissues and cells. Sufferers with high expression of LINC00284 possessed a larger tumor size, but it’s worth noting that the sample size of PTC patients included in this research was comparatively small, which might lead to low statistical power. This was a possible reason that we didn’t detect the association between LINC00284 expression and other clinicopathological characteristics including extrathyroidal extension, lymph node metastasis, multifocality, or TNM stage. In the future, we will further collect more PTC patients to explore the clinical significance of LINC00284. Considering that LINC00284 was correlated to tumor size, we explored the impacts of LINC00284 on the proliferation ability of PTC cells. In line with Ruan et al.’s study, our results also identified that silencing LINC00284 could suppress PTC cell proliferation in vivo and in vitro, additionally, induce G0/G1 arrest and apoptosis. Nevertheless, our study also existed a certain limitation, only evaluated whether LINC00284 impacted PTC cells growth according to the results of clinicopathologic data analyses, not involving in relevant detections of migration and invasion. So, more efforts need to be put into analyze the effects of LINC00284 on other PTC cell processes. No matter how these results revealed that LINC00284 could function as an oncogene in PTC.

There is plenty of evidence that lncRNAs could serve as miRNA sponges, attenuating miRNA-mediated the inhibition of their target mRNAs [9, 22]. Our study firstly predicted that most of LINC00284 exerted in the cytoplasm of PTC cells, which was confirmed by FISH and subcellular fractionation assays and determined the possibility of LINC00284 regulating gene expression at the post-transcriptional level. The subsequent bioinformatics analyses and dual-luciferase reporter assay suggested that LINC00284 could bind to miR-3127-5p. Moreover, we conducted Spearman’s correlation analysis and found that the expression of LINC00284 was negatively related to miR-3127-5p in 75 PTC samples. According to previously published literature, miR-3127-5p was known as a tumor suppressor in various cancers. In non-small-cell lung cancer, reduction of miR-3127-5p accelerated EMT via activating the Wnt/FZD4/β-catenin signaling pathway [28]. Overexpressing miR-3127-5p could inhibit cell growth and induce apoptosis in glioma [29]. Likewise, our results showed that miR-3127-5p was decreased in PTC tissues and reducing its expression promoted PTC cell proliferation. The subsequent rescue experiments indicated that the suppression of cell growth caused by silencing LINC00284 was partially abolished by a miR-3127-5p inhibitor. To sum up, LINC00284 acted as a sponge of miR-3127-5p and its function is mediated in part by negative modulation of miR-3127-5p.

Previous reports have proven that miRNAs played a key role in various biological processes of PTC by targeting relevant mRNAs [30, 31]. In our study, three online prediction tools were adopted to search the targets of miR-3127-5p and 130 mRNAs were preliminarily identified. Next, seven candidate genes were picked out from the total because they exhibited higher expression (fold change ≥ 2, *P* < 0.05) in TC samples than normal samples via analyzing TCGA data, followed by western blot assays. Finally, we selected E2F7 as the target mRNA of miR-3127-5p due to the most obvious increase of E2F7 protein levels by miR-3127-5p inhibitor. The following luciferase reporter assay confirmed the binding of miR-3127-5p and E2F7. In addition, further qRT-PCR and western bolt showed that E2F7 could be directly inhibited by miR-3127-5p and indirectly enhanced by LINC00284 at both the mRNA and protein levels. Recently, E2F7 has been reported to be closely related to drug resistance, cell cycle, and DNA damage repair in several cancers [32, 33]. Similarly, our study also highlighted the high levels of E2F7 in PTC tissues and the relationship of its high expression with poor disease-free survival of TC patients from TCGA, while the silencing of E2F7 resulted in a decrease in PTC cell proliferation. Rescue assays revealed that the facilitation effects on PTC cell proliferation arisen from silencing miR-3127-5p could be reversed by the repression of E2F7 in part, suggesting that miR-3127-5p inhibited PTC cell growth based on reducing E2F7 expression.

In summary, we first determined that lncRNA LINC00284 was an oncogene that accelerated cell proliferation in PTC and identified a novel ceRNA network that LINC00284 increased E2F7 expression via sponging miR-3127-5p (Fig. 8). Hence, LINC00284 might become a potential useful diagnostic and therapeutic target for PTC.

## Materials and methods

### Tissue samples

We gathered paired PTC samples and matched adjacent non-tumor samples from 75 patients, who received surgery at the Affiliated Jiangyin Hospital of Southeast University (Wuxi, China) during 2016 and 2019. Before the operation, none of these subjects received any chemotherapy or radiotherapy. All tissue specimens were promptly snap frozen in liquid nitrogen and stored at −80 °C for mRNA or protein extraction. The study got access to the informed consent of all included participants and was simultaneously approved by the Research Ethics Committee of The Affiliated Jiangyin Hospital of Southeast University Medical College.

### Cell culture and transfection

Three PTC cell lines (IHH-4, TPC-1, and K-1) and a normal thyroid follicular epithelium cell line (Nthy-ori3-1) were gifts from the laboratory of General Surgery, Jiangsu Province Hospital. All cells were tested for mycoplasma contamination and authenticated by Short Tandem Repeat profiling recently. We used RPMI 1640 medium (Hyclone, USA) containing ten percent fetal bovine serum (FBS) (Clark, USA) to culture K-1 and Nthy-ori3-1 cells. TPC-1 cells were kept in DMEM high glucose (Hyclone, USA) supplemented with 15% FBS. IHH-4 was cultured in a mixture (1:1) of DMEM supplemented with ten percent FBS and RPMI 1640. We added 1% antibiotics containing 100 mg/ml streptomycin and 100 units/ml penicillin into the above two mediums. These cell lines were incubated at thirty-seven degrees Celsius with 5% CO_2_ in a humidified atmosphere. We entrusted the GenePharma Company (Shanghai, China) to design and synthesize the relevant short interfering RNA (siRNA) (si-LINC00284 and si-E2F7), short hairpin RNA (shRNA) (shLINC00284), miR-3127-5p mimics or inhibitor, and corresponding negative controls (NC). Following the manufacturer’s protocols, all transfections were carried out employing Lipofectamine 3000 (Invitrogen). We added 2 mg/ml puromycin into mediums to single out stably transfected cells.

### Quantitative real‐time polymerase chain reaction (qRT-PCR)

We extracted total RNA from tissues and cells employing TRIzol reagent (Invitrogen, Carlsbad, California). Then the quality and concentration of RNA were detected by NanoDrop spectrophotometer (ND-100, Thermo). RNA (1 μg) was reverse transcribed to complementary DNA (cDNA) through utilizing the PrimeScript RT Master Mix Kit (TaKaRa, Japan). We applied the ReverAid Transcriptase Kit (Thermo Scientific, USA) to conduct the reverse transcription for miR-3127-5p. The qRT-PCR was used to determine the expression levels of LINC00284, miR-3127-5p, and E2F7 with the SYBR Green Master Mix kit (TaKaRa, Japan) on a 7500 Real-time PCR System. The qRT-PCR results were showed with the 2^−ΔΔCt^ method and normalized to GAPDH or U6. The primers sequences as follows: LINC00284:

5′-CCAGGGGATAAAACCCGCTT-3′ (forward), 5′-TAAGCACCAAGTCACGCTGT-3′ (reverse), E2F7: 5′-GGTCAGGGTCAGAGAGGGAT-3′ (forward); 5′-GACCATGCAAGGGACACTGA-3′ (reverse), GAPDH 5′-CACCCACTCCTCCACCTTTG-3′ (forward), 5′-CCACCACCCTGTTGCTGTAG-3′ (reverse).

### Western blot analysis

The RIPA buffer (Beyotime, Shanghai, China) was applied to harvested and extracted total protein from TPC-1 and K-1 cells, whose concentration was subsequently analyzed by BCA Protein Detection Kit (Pierce, Appleton, WI, USA). Cell protein lysates were subjected to SDS-PAGE gel, subsequently transferred to polyvinylidene fluoride (Millipore) membranes. Before the membranes were incubated with specific primary antibodies at four degrees Celsius overnight, they were blocked in Tris-buffered saline and Tween-20 containing 5% nonfat powdered milk for 2 h. Following washing four times using TBST, the membranes were incubated with matched secondary antibodies. The protein bands were visualized by an enhanced chemiluminescence kit (Thermo Fisher). We employed GAPDH as the internal reference. Primary antibodies against CDK4 (cat. 11026-1-AP), Cyclin D3 (cat. 26755-1-AP), Bcl-2 (cat. 12789-1-AP), GAPDH (cat. 10494-1-AP), TNNI1 (cat. 16102-1-AP), ETV5 (cat. 13011-1-AP), E2F7 (cat. 24489-1-AP), and MISP (cat. 26338-1-AP) were purchased from Proteintech (Wuhan, China). Besides, we used primary antibodies against Bak (cat. 12105, CST, Danvers, MA, USA), CLIP3 (cat. ab74239, Abcam, Cambridge, UK), ACOT12 (cat. PA5–32152, Invitrogen, California, USA), and FAXC (cat. bs-15229R-1, Hengfei, Shanghai, China).

### Cell proliferation assays

For CCK-8 assay, we seeded TPC-1 and K-1 cells after transfection in 96-well plates (1000 cells/well) and cultured them at 37 °C with five percent CO_2_. The cell viability was examined every 24 h, in brief, 10 μL CCK8 solution was added into per well. After incubation for 2 h at 37 °C, the microplate reader was utilized to detect the absorbance of all wells at an optical density of 450 nm. We planted 500 cells in 6-well plates and cultured them at 37 °C with 5% CO_2_ for 14 days in colony formation assay. Following being washed, the colonies were fixed in four percent paraformaldehyde for fifteen minutes, dyed by 0.1% crystal violet solution for 20 min, and finally counted manually. For Ethynyldeoxyuridine (EdU) assay, we seeded the transfected cells (density: 5000 cells/well) on ninety-six-well plates. Then, 50 μM EdU labeling medium (RiboBio, Guangzhou, China) was added to these wells and then incubated for 2 h under 5% CO_2_ at 37 °C. After treated with four percent paraformaldehyde and 0.5% Triton X-100, cells were stained by Apollo Dye Solution. Then Hoechst 33342 was used to stain the nucleic acids within the cells. Ultimately, we used fluorescence microscopy (Nikon, Japan) to analyze and calculate the rate of EdU-positive cells.

### Flow cytometric analysis

For cell cycle assay, the transfected cells were dyed by propidium iodide (PI) (MULTI SCIENCES, Zhejiang, China). Then we used flow cytometry to count and compare the number of G0/G1, S, and G2/M phase cells. In the apoptosis assay, after double stained with FITC-Annexin V and PI with the AnnexinV-FITC/PI Apoptosis Detection Kit (Beijing Biosea Biotechnology, China), the treated TPC-1 and K-1 cells were divided into four categories, that is viable, dead, early apoptotic, and terminal apoptotic cells. The apoptotic rate was defined as the proportion of early and late apoptotic cells.

### Animal experiments

We purchased 30 female BALB/c nude mice (3–4 weeks old) from Southeast University Animal Center, and gained the approval of the Institutional Animal Care and Use Committee. These mice were randomly allocated to two groups (TPC-1-NC and TPC-1-shLINC00284). After being resuspended in 100 µl PBS, 2 × 10^6^ TPC-1 cells transfected stably with LINC00284 shRNA or control vector were subcutaneously inoculated into the flank of nude mice. The tumor size of xenografts was measured every three days following the formula: *V* = 0.5 × *D* × *d*^2^ (*V*, volume; *D*, longitudinal diameter; *d*, latitudinal diameter). After fifteen days, we euthanized these mice, then excised and weighted the implanted tumors. The investigators were not blinded to the group allocation during the experiment and when assessing the outcome.

### Immunohistochemistry staining

The xenograft tissues resected from nude mice were fixed in four percent paraformaldehyde, dehydrated, and embedded in paraffin. We cut the specimens into 4-mm-thick sections, which were then deparaffinized with xylenes and rehydrated by graded ethanol washes. Next, the sections were processed with specific primary antibodies Ki-67 (cat. 27309-1-AP, Proteintech, Wuhan, China) and Cleaved Caspase-3 (cat. 9661, CST, Danvers, MA, USA) at 4 °C overnight, subsequently incubated with secondary antibodies at room temperature for 30 min. 3,3-diaminobenzidine solution was employed to incubate the slides for visualization. Hematoxylin was applied for counterstaining. Finally, these slides were observed and photographed using a microscope.

### FISH and subcellular fractionation assays

Ribobio (China) Company was commissioned to design and synthesize the LINC00284 FISH probes, which were then labeled with Cy3 fluorescent dye. 2 × 10^4^ PTC cells were planted in a confocal dish for one day, followed being fixed and permeabilized. Then we blocked these cells with 250 µl prehybridization agent, mixed with hybridization buffer containing 20 μm probe and added them into the dishes for hybridizing with target sequence. After staining the nuclei with DAPI, the fluorescent images were photographed and recorded under a LSM5 Live confocal microscope (Carl Zeiss AG, Jena, Germany). The Cytoplasmic and Nuclear RNA Purification kit (Norgenbiotek Corporation, Canada) was used to separate and purify cytoplasmic and nuclear RNA of TPC-1 and K-1 cells following the manufacturers’ description. Then we performed qRT-PCR to detect the subcellular fractions.

### Dual-luciferase reporter assay

The LINC00284 or E2F7 fragments containing mutated or wild-type miR-3127-5p binding sites were synthesized and subcloned into the pmirGLO luciferase reporter vector (Promega, Madison, WI, USA). After HEK-293T cells being implanted in 24-well plates for one day, we cotransfected the luciferase reporter vectors and miR-3127-5p mimics or control vector into HEK-293T cells. Forty-eight hours after transfection, the Dual-Luciferase Kit (Promega) was used to analyze the relative luciferase activities, which were normalized to renilla luciferase activity.

### Bioinformatic analysis and statistical analysis

LncRNASNP2 (http://bioinfo.life.hust.edu.cn/lncRNASNP/) and RegRNA 2.0 (http://regrna2.mbc.nctu.edu.tw/index.html) were applied to predict the downstream miRNA targets of LINC00284. The targets of miR-3127-5p were searched through Targetscan (http://www.targetscan.org/vert_72/), DIANA (http://diana.imis.athena-innovation.gr/DianaTools), and miRDB (http://mirdb.org/cgi-bin/). We downloaded two datasets named TCGA-THCA.htseq_fpkm-uq.tsv (comprising 510 PTC samples and 58 normal samples) and TCGA-THCA.mirna.tsv (including 514 PTC samples and 59 normal samples) from the UCSC cancer browser with version number 07–20–2019 (https://xenabrowser.net/datapages/). The standardized expression values of LINC00284, miR-3127-5p, and E2F7 were obtained from the above files. Furthermore, the GSE66783 [34] dataset (containing 5 paired PTC samples and adjacent normal samples) from NCBI GEO (https://www.ncbi.nlm.nih.gov/gds/) was also used for assessing the expression of LINC00284 and E2F7. The online tool, lncLocator (http://www.csbio.sjtu.edu.cn/bioinf/lncLocator/), was performed to analyze the subcellular localization of LINC00284. We acquired the survival data of E2F7 from the Gene Expression Profiling Interactive Analysis (GEPIA, http://gepia.cancer-pku.cn/). The significance of differences was calculated via *χ*^2^ test and Student’s *t* test (data met normal distribution and homogeneity of variance, otherwise the Wilcoxon rank sum test was used). The data were exhibited as the mean ± standard errors or standard deviation unless otherwise specified. The GraphPad Prism software version 6.0 and Social Sciences (SPSS) software version 25.0 were utilized to conduct all statistical analyses. All assays were repeated independently at least three times. *P* values <0.05 determined statistical significance (*P < 0.05, **P < 0.01, ***P < 0.001).

## Supplementary information

Supplementary Figure 1

## References

[1] Siegel RL, Miller KD, Jemal A (2019). Cancer statistics, 2019. CA Cancer J. Clin..

[2] Xing M (2013). Molecular pathogenesis and mechanisms of thyroid cancer. Nat Rev Cancer.

[3] Hartl DM, Hadoux J, Guerlain J, Breuskin I, Haroun F, Bidault S (2019). Risk-oriented concept of treatment for intrathyroid papillary thyroid cancer. Best Pr Res Clin Endocrinol Metab..

[4] Lubitz CC, Sosa JA (2016). The changing landscape of papillary thyroid cancer: epidemiology, management, and the implications for patients. Cancer..

[5] Lee YC, Na SY, Park GC, Han JH, Kim SW, Eun YG (2017). Occult lymph node metastasis and risk of regional recurrence in papillary thyroid cancer after bilateral prophylactic central neck dissection: a multi-institutional study. Surgery..

[6] Djebali S, Davis CA, Merkel A, Dobin A, Lassmann T, Mortazavi A (2012). Landscape of transcription in human cells. Nature..

[7] Consortium EP, Birney E, Stamatoyannopoulos JA, Dutta A, Guigo R, Gingeras TR (2007). Identification and analysis of functional elements in 1% of the human genome by the ENCODE pilot project. Nature..

[8] Morlando M, Fatica A (2018). Alteration of epigenetic regulation by long noncoding RNAs in cancer. Int J Mol Sci.

[9] Dai G, Huang C, Yang J, Jin L, Fu K, Yuan F (2020). LncRNA SNHG3 promotes bladder cancer proliferation and metastasis through miR-515-5p/GINS2 axis. J Cell Mol Med.

[10] Dong Z, Gao M, Li C, Xu M, Liu S (2020). LncRNA UCA1 antagonizes arsenic-induced cell cycle arrest through destabilizing EZH2 and facilitating NFATc2 expression. Adv. Sci..

[11] Lan Z, Yao X, Sun K, Li A, Liu S, Wang X (2020). The Interaction between lncRNA SNHG6 and hnRNPA1 contributes to the growth of colorectal cancer by enhancing aerobic glycolysis through the regulation of alternative splicing of PKM. Front Oncol..

[12] Xu J, Lu Y, Liu Q, Xia A, Zhao J, Xu X (2020). Long noncoding RNA GMAN promotes hepatocellular carcinoma progression by interacting with eIF4B. Cancer Lett..

[13] Han T, Wu Y, Hu X, Chen Y, Jia W, He Q (2020). NORAD orchestrates endometrial cancer progression by sequestering FUBP1 nuclear localization to promote cell apoptosis. Cell Death Dis..

[14] Nie J, Zhao Q (2020). Lnc-ITSN1-2, derived from RNA sequencing, correlates with increased disease risk, activity and promotes CD4(+) T cell activation, proliferation and Th1/Th17 cell differentiation by serving as a ceRNA for IL-23R via sponging miR-125a in inflammatory bowel disease. Front. Immunol..

[15] Chen S, Wang G, Tao K, Cai K, Wu K, Ye L (2020). Long noncoding RNA metastasis-associated lung adenocarcinoma transcript 1 cooperates with enhancer of zeste homolog 2 to promote hepatocellular carcinoma development by modulating the microRNA-22/Snail family transcriptional repressor 1 axis. Cancer Sci..

[16] Vidovic D, Huynh TT, Konda P, Dean C, Cruickshank BM, Sultan M (2020). ALDH1A3-regulated long non-coding RNA NRAD1 is a potential novel target for triple-negative breast tumors and cancer stem cells. Cell Death Differ..

[17] Ruan Z, Zhao D (2019). Long intergenic noncoding RNA LINC00284 knockdown reduces angiogenesis in ovarian cancer cells via up-regulation of MEST through NF-kappaB1. FASEB J..

[18] Xu J, Zhang J, Shan F, Wen J, Wang Y (2019). SSTR5AS1 functions as a ceRNA to regulate CA2 by sponging miR15b5p for the development and prognosis of HBVrelated hepatocellular carcinoma. Mol Med Rep..

[19] Wang C, Li Y, Yan S, Wang H, Shao X, Xiao M (2020). Interactome analysis reveals that lncRNA HULC promotes aerobic glycolysis through LDHA and PKM2. Nat. Commun..

[20] Zhang Q, Li T, Wang Z, Kuang X, Shao N, Lin Y (2020). lncRNA NR2F1-AS1 promotes breast cancer angiogenesis through activating IGF-1/IGF-1R/ERK pathway. J Cell Mol Med.

[21] Tang T, Guo C, Xia T, Zhang R, Zen K, Pan Y (2019). LncCCAT1 promotes breast cancer stem cell function through activating WNT/beta-catenin signaling. Theranostics..

[22] Wu DM, Wang S, Wen X, Han XR, Wang YJ, Shen M (2018). LncRNA SNHG15 acts as a ceRNA to regulate YAP1-Hippo signaling pathway by sponging miR-200a-3p in papillary thyroid carcinoma. Cell Death Dis..

[23] Wen J, Chen L, Tian H, Li J, Zhang M, Cao Q (2019). Effect of MALAT1 polymorphisms on papillary thyroid cancer in a chinese population. J Cancer.

[24] Wen J, Wang H, Dong T, Gan P, Fang H, Wu S (2019). STAT3-induced upregulation of lncRNA ABHD11-AS1 promotes tumour progression in papillary thyroid carcinoma by regulating miR-1301-3p/STAT3 axis and PI3K/AKT signalling pathway. Cell Prolif..

[25] Zhang Y, Hu J, Zhou W, Gao H (2018). LncRNA FOXD2-AS1 accelerates the papillary thyroid cancer progression through regulating the miR-485-5p/KLK7 axis. J Cell Biochem.

[26] Ye Y, Song Y, Zhuang J, He S, Ni J, Xia W (2018). Long non-coding RNA CCAL promotes papillary thyroid cancer progression by activation of NOTCH1 pathway. Oncol Res..

[27] Xing C, Cai Z, Gong J, Zhou J, Xu J, Guo F (2018). Identification of potential biomarkers involved in gastric cancer through integrated analysis of non-coding RNA associated competing endogenous RNAs network. Clin Lab.

[28] Yang Y, Sun Y, Wu Y, Tang D, Ding X, Xu W (2018). Downregulation of miR-3127-5p promotes epithelial-mesenchymal transition via FZD4 regulation of Wnt/beta-catenin signaling in non-small-cell lung cancer. Mol Carcinog..

[29] Pan B, Zhao M, Xu L (2019). Long noncoding RNA gastric cancer-associated transcript 3 plays oncogenic roles in glioma through sponging miR-3127-5p. J Cell Physiol..

[30] Yu S, Cao S, Hong S, Lin X, Guan H, Chen S (2019). miR-3619-3p promotes papillary thyroid carcinoma progression via Wnt/beta-catenin pathway. Ann Transl Med.

[31] Zhao X, Li Y, Zhou Y (2019). MicroRNA-96-3p promotes metastasis of papillary thyroid cancer through targeting SDHB. Cancer Cell Int.

[32] Lu C, Wei Y, Wang X, Zhang Z, Yin J, Li W (2020). DNA-methylation-mediated activating of lncRNA SNHG12 promotes temozolomide resistance in glioblastoma. Mol Cancer.

[33] Mitxelena J, Apraiz A, Vallejo-Rodríguez J, García-Santisteban I, Fullaondo A, Alvarez-Fernández M (2018). An E2F7-dependent transcriptional program modulates DNA damage repair and genomic stability. Nucleic Acids Res.

[34] Lan X, Zhang H, Wang Z, Dong W, Sun W, Shao L (2015). Genome-wide analysis of long noncoding RNA expression profile in papillary thyroid carcinoma. Gene..

