# Supplementary figures and images for: Long noncoding RNA LINC00284 facilitates cell proliferation in papillary thyroid cancer via impairing miR-3127-5p targeted E2F7 suppression
Source: Cell Death Discov. 2021 Jun 26;7:156. doi: 10.1038/s41420-021-00551-8 (PMC8257569; doi:10.1038/s41420-021-00551-8)

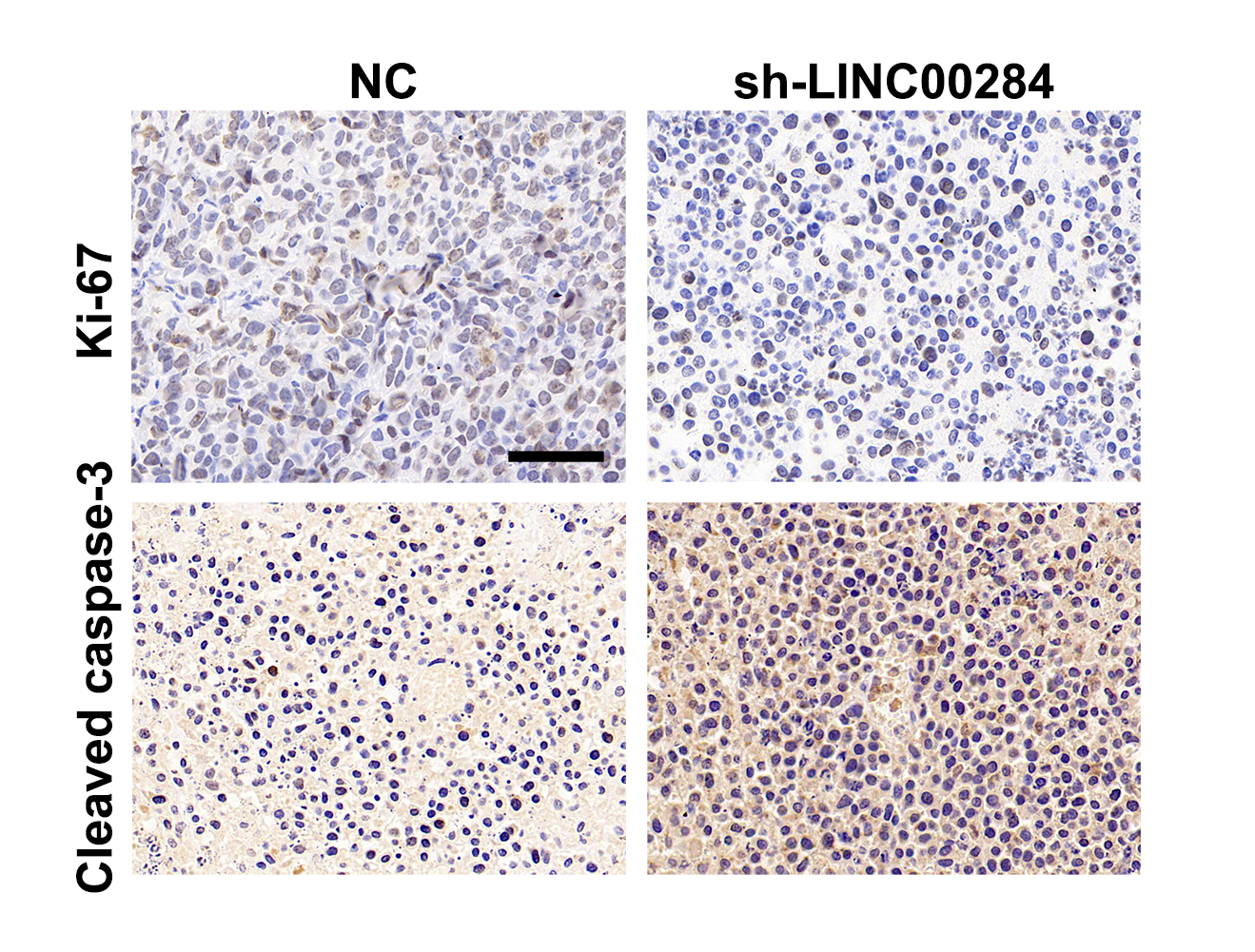

Supplement: Supplementary file 1 — Supplementary Figure 1 [file 41420_2021_551_MOESM1_ESM.tif]
